# Causal Relationships Between Physical Activity and Arthrokinematic Integrity of the Ankle Joint-Foot Complex Across Normal and Pathological Phenomena: A Case-Control Analysis

**DOI:** 10.7759/cureus.59578

**Published:** 2024-05-03

**Authors:** Sundip Charmode, Simmi Mehra, Abhishek Kumar Mishra

**Affiliations:** 1 Anatomy, All India Institute of Medical Sciences (AIIMS) Rajkot, Rajkot, IND; 2 Orthopedics and Trauma, All India Institute of Medical Sciences (AIIMS) Rajkot, Rajkot, IND

**Keywords:** lack of physical activity, ankle joint -foot, painful pathologies, original study, arthrokinematics

## Abstract

Introduction

Muscles, ligaments, tendons, bones, and cartilage undergo age-related changes, affecting the foot-ankle joint complex biomechanics in both genders. While international studies have extensively researched these dynamics, Indian studies are limited. Our study aims to fill this gap by analyzing the anthropometric and biomechanical function of the foot-ankle joint complex in normal individuals and those with painful pathologies at All India Institute of Medical Sciences (AIIMS) Rajkot’s OPD.

Methods

In a two-year case-control study of the cross-sectional type conducted at AIIMS Rajkot’s OPD, 158 patients with similar pain intensity on the Numeric Pain Rating Scale were examined. Anthropometric and biomechanical measurements were taken for both affected and non-affected foot and ankle joints. Cases comprised patients with foot and ankle joint pain, while controls were selected based on predefined criteria and were without such pain. Ethical approval was acquired from the Institutional Ethical Committee of AIIMS Rajkot.

Results

The sprain of the ankle joint and foot was the most common musculoskeletal pathology (65 out of 158 cases, i.e., 41.13%) affecting the ankle joint-foot complex. Patients involved in occupations requiring higher physical inactivity suffer more commonly from ankle joint-foot pathologies. The mean difference in the range of motion, i.e., dorsiflexion, plantar flexion, inversion, and eversion, between affected and non-affected feet was found to be lower in the patients who belonged to occupations involving low physical activity compared to those patients having occupations with high physical activity.

Conclusion

Reduced physical activity increases the stiffness and reduces the flexibility of the tendons, muscles, and ligaments of any joint (the ankle joint-foot complex in this study) and is associated with a higher incidence of musculoskeletal pathologies.

## Introduction

Muscle, ligament, tendon, bone, and cartilage undergo morphological and physiological changes with aging in both genders, impacting the biomechanical function of the foot-ankle joint complex in normal and pathological conditions [1]. Brockett and Chapman [1], Medina McKeon et al. [2], and Amaha et al. [3] are a few notable researchers who substantially studied the biomechanics and kinesiology of the foot-ankle joint complex in normal as well as arthritic patients. In India, Kumar et al. [4] studied the range of motion (ROM) of hip and ankle joints in the normal Indian population between the ages of one month and 75 years. Kharat et al. [5] evaluated the use of MRI in diagnosing ankle joint and foot pathologies, whereas Sabharwal and Singh [6] studied the prevalence of ankle instabilities and disabilities among female Kathak dancers. In the Indian population, no study exists to date that analyzes the biomechanical function of the foot-ankle joint complex in normal patients and patients with painful pathologies. Hence, a long-term, observational case-control study is required. The anthropometric and biomechanical data arising from such an analytical study will enable investigators and physicians to identify and record high-risk patients and analyze the influence of physical activity involved in their occupation on their ability to regain their full functional ability by the end of the rehabilitation phase. The database will also be utilized for treating patients with documented ankle joint-foot complex pathologies in the future, focusing on Phase 2 of our project, which employs gait modification techniques.

## Materials and methods

Study design and population

We conducted a case-control study of cross-sectional type in the OPD of the Department of Orthopedics, All India Institute of Medical Sciences (AIIMS) Rajkot, and the anthropometry laboratory of the Department of Anatomy, AIIMS Rajkot. The study duration was two years after approval from the Institutional Ethical Committee (IEC) of AIIMS Rajkot. Clinical Trials Registry - India (CTRI) registration was done prospectively, and the number and date are CTRI/2023/10/058206, October 3, 2023. A sample size of 240 patients (including 120 cases suffering from foot and ankle joint pain and 120 controls currently not suffering from foot and ankle joint pain) was calculated. There was no previous or reference study available on the same topic conducted among the Indian population. So, to calculate the sample size, the population prevalence of the ankle joint-foot pathologies was required to be estimated. The statistical data on total patients reporting to the OPD of the orthopedic department of AIIMS Rajkot was obtained at the end of the first year of study (i.e., in the period between August 1, 2022, and July 31, 2023).

Sample size

The statistical data was collected after obtaining prior permission from the competent authority of AIIMS Rajkot. The sample size was calculated based on a reference study by Keenan et al. that was conducted in the British population, in which the prevalence and impact of self-reported foot and ankle pain in the over-55 age group were observed to be 18.4% [7]. Considering n as the sample size for the study group, P was 18.4, Q was 81.6, L was taken as 5% with a 95% CI, and the power of the study was considered as 80.0. Using the formula, sample size (n) = Z^2^ x PQ/L^2^, n was calculated as 230.68, which was rounded off to 240. So, the sample size (n) amounted to 120 cases and equal controls. To support this calculated sample size, the statistical data of the institute during the first one-year duration of the study, i.e., from August 1, 2022, to July 31, 2023, was taken. Out of the total of 10,679 patients reporting to the OPD of the orthopedic department of AIIMS Rajkot, around 263 patients belonged to ankle joint-foot complex pathology. An approximate incidence rate of 2.46% was observed. Finally, based on feasibility, a sample size of 240 with 120 cases and equal controls was finalized for this study.

Inclusion and exclusion criteria

Eligibility criteria (inclusion and exclusion) for both case and control patients were framed by the investigators. Those patients (both case and control) who satisfied the inclusion criteria and exclusion criteria and who gave written consent were selected as study participants. The inclusion criteria for the case were the following: (i) participants should be natives of any region of the country and (ii) they should present with foot and ankle joint pain of musculoskeletal origin. The exclusion criteria for the case were the following: (i) should be within the normal ranges of BMI; (ii) should not be more than 70 years and less than 14 years of age; (iii) should not have undergone either partial or complete joint transplant; (iv) should not have any congenital or acquired deformity or metabolic disorders or malignancy, pregnancy, or any other debilitating disease; (v) should not have a history of fracture of foot and ankle joint; and (vi) should not have a history of any surgery performed on foot and ankle joints.

The inclusion criteria for control were the following: (i) participants native to any region of the country and (ii) the current physical condition of the foot and ankle joint should be pain-free. The exclusion criteria for control were the following: (i) should be within the normal ranges of BMI; (ii) should not be more than 70 years and less than 14 years of age; (iii) should not have undergone partial or complete joint transplant or use walking aids; (iv) should not have any congenital or acquired deformity or metabolic disorders or malignancy, pregnancy, or any other debilitating disease; (v) should not have a history of fracture of foot and ankle joint; and (vi) should not have a history of any surgery performed on ankle joint and foot. One of the coinvestigators was an orthopedic surgeon, who was the first point of contact with the patients. The patients were screened for the eligibility criteria at the orthopedic OPD using their history, investigations, and radiological findings, and once they satisfied the criteria, informed consent was offered.

Ethical approval

This study was conducted as Phase 3 of an intramural research project (non-funded) in the Department of Anatomy in collaboration with the Department of Orthopedics at AIIMS Rajkot. The project was submitted and presented to the Research Review Board, and after its approval, it was forwarded to the IEC of the AIIMS, Rajkot, Gujarat-360110. The IEC approval was received on July 9, 2022, with the protocol ID NF/15/2022. The approval letter with reference number O.W.No./AIIMS. Rajkot/IEC/13/2022, dated July 9, 2022, has been issued.

CTRI registration

After the IEC approval, the project was submitted to the CTRI, and registration was successfully completed on October 3, 2023. The registration number is CTRI/2023/10/058206.

Patient information sheet or data collection form

To collect general information about the participant, a specially designed patient information form (in English, Hindi, and Gujarati) was formulated to collect demographic data (age, gender, religion, occupation, education, contact details, and postal address), past medical history, past surgical history of the concerned party, present diagnosis, mode of treatment received (conservative or surgical) for the presenting condition (in the past and now), unilateral or bilateral involvement, and duration of the illness of the participants. An informed consent proforma was prepared and given separately along with the patient information sheet, and consent was obtained for participating in the study.

Data collection procedure

The anthropometric measurements of the foot were taken as follows [8]. The height/stretch stature was measured using a stadiometer as the perpendicular distance between the transverse planes of the vertex and the inferior aspect of the feet. The person stood in the anatomical position with feet together, the back in contact with the stadiometer, and the head in the Frankfort plane. For measuring the weight or body mass, the person was made to stand on a digital weighing machine in an anthropometric position, and the mass or weight was calculated as the force the mass exerted in a standard gravitational field. The BMI was calculated using the formula: BMI is weight in kilograms divided by height in meters squared. If height has been measured in centimeters, it is divided by 100 to convert it to meters [9]. Foot length (FL) was measured as the distance between the posterior-most point and the anterior-most point of the foot. The foot breadth (FB) was measured as the distance between the point projecting most medially on the head of the first metatarsal bone and the point projecting most laterally on the head of the fifth metatarsal bone. The truncated FL (TFL) was measured as the distance between the most rear load support portion from the calcaneus to the first metatarsal head [10]. Medial longitudinal arch height (MLAH) was measured as the perpendicular distance from navicular tuberosity to the TFL [11]. The longitudinal arch angle (LAA) was calculated by drawing a line from the center of the medial malleoli to the navicular tuberosity, and another line was drawn from the navicular tuberosity to the head of the first metatarsal. The obtuse angle between these lines was measured as LAA [5]. The arch height index was measured as the height of the dorsum of the foot at 50% of the FL divided by the TFL [11]. The Feiss line was measured as a line that was drawn from the center of the medial malleoli to the head of the first metatarsal [11].

For calculating the biomechanical measurements, i.e., the ROM at the ankle joint-foot complex, a manual (non-digital) goniometer was used at the ankle joint-foot complex, as it is the gold standard with inter- and intra-rater reliability almost equivalent to that of the dual-axis inclinometer [12-15]. At the ankle joint, dorsiflexion and plantar flexion were measured. At the subtalar joint, inversion and eversion were measured.

For measuring the dorsiflexion, the patient was made to lie supine, extending his or her knee, and the subtalar joint was stabilized in a neutral position. Dorsiflexion at the ankle was tried by pushing through the fifth metatarsal head. The goniometer was aligned in such a way that the axis passed through the lateral malleolus, the stationary arm was aligned with the fibular head, and the moving arm was aligned with the fifth metatarsal. For measuring the plantar flexion, the patient was made to lie supine with the knee extended and the ankle in an anatomical position. The leg was stabilized. The ankle was plantar flexed. The goniometer alignment was in such a way that the axis passed through the lateral malleolus, the stationary arm was aligned with the fibular head, and the moving arm was aligned with the fifth metatarsal. For measuring the inversion at the subtalar joint, the patient was made to lie prone. The tibia was stabilized in the sagittal plane (rotate the hip or pelvis to align the tibia). Calcaneus was inverted. The goniometer was aligned such that the stationary arm was aligned with the midline of the leg and the moving arm was aligned with the midline of the calcaneus. For measuring the eversion at the subtalar joint, the patient was made to lie prone. The tibia was stabilized in the sagittal plane (rotate the hip or pelvis to align the tibia). Calcaneus was everted. The goniometer was aligned such that the stationary arm was aligned with the midline of the leg and the moving arm was aligned with the midline of the calcaneus. The standard error of the stadiometer is between 0.4 and 0.5 cm. This error was even after measurements were done by only a single person every time. The standard error of a manual goniometer is between 2.4 and 4.9 degrees; on repeated measurements, that was less than 5 degrees, which was acceptable in a large sample-sized study.

Data categorization

The data collected from the study participants was categorized into high occupational activity (OA) and low OA groups. For classification based on physical activity, the Alphabetical Indexes of Industries and Occupations issued by the United States Census Bureau 1980 Report was used [16]. Based on the amount of physical activity involved in the occupation, the occupations were classified as those involving high physical activity, which included laborers, farmers, etc. Another group was those occupations involving low physical activity, including doctors, engineers, executives, etc. [17].

Risk assessment

The study participants, after satisfying the eligibility criteria, were assessed for pain intensity using the Numeric Pain Rating Scale and categorized into mild, moderate, and worst pain. The study participant was asked to rate the pain he or she had experienced over the past 24 hours. Such three pain ratings correspond to the current, best, and worst pain experienced. The average of the three ratings was taken and used to represent the patient’s level of pain over the previous 24 hours [18]. Intra-subject variability in the ROM at the ankle joint-foot complex was examined; in cases of variation observed beyond the normal ranges of motion in the unaffected pain-free foot, previously conducted radiographs of the said foot and ankle joint were used to measure the ROM at the bony angles. A pilot study was conducted on 100 participants during the first 12 months of the project, from August 1, 2022, until July 30, 2023, and the intra-rater and inter-rater reliability of the measurement of ROM was determined to be satisfactory on both affected and non-affected sides.

The inter-rater reliability was preserved by conducting the measurements of all 158 patients (on both the affected and non-affected sides) by two of the authors (principal investigator and coinvestigator -01) separately. At the end of every five cases, both investigators reviewed and matched their measurements, and in cases where more than a 5% difference was observed, the third coinvestigator was involved in taking the measurements and comments. Intra-observer variation was made by making sure that each of the investigators who collected the data took three readings and took the average of them. This was done in all the cases.

Data collection instruments

Anthropometry instruments, including digital vernier calipers, stadiometers, goniometers (0-180 degrees and 0-360 degrees), digital weight machines, skin marking pens, plastic rulers, triangular scale ruler sets, measuring tape, patient information forms, and stationary, were required for data collection.

Data analysis procedure

The collected data was compiled in an Excel sheet, a master chart was prepared, and accordingly, tables and graphs were prepared. The name of the patient was kept anonymous in the study. For quantitative data analysis (of every variable), the mean and standard deviation were calculated, and the P-value was determined. If the P-value was less than 0.05, in that case, it was considered significant. All the quantitative data (foot anthropometric and biomechanical variables like FL, FB, TFL, LAA, MLAH, dorsiflexion, plantar flexion, inversion, and eversion) was compared on the right and left sides, male and female. A sample t-test was used, and a P-value was determined. All the quantitative data (foot anthropometric and biomechanical variables like FL, FB, TFL, LAA, MLAH, dorsiflexion, plantar flexion, inversion, and eversion) were compared in three groups (young, adult, and older age groups). The ANOVA test was used, and the P-value was determined. The statistical data was analyzed by IBM SPSS Statistics for Windows, Version 25.0 (Released 2017; IBM Corp., Armonk, NY, USA). The data was tabulated and graphically represented after analysis.

## Results

The project was divided into an initial pilot period, followed by a continuation period. The objective of the 12-month pilot phase (from August 1, 2022 to July 30, 2023) was to estimate the sample size required for the study by identifying and registering the patients reporting to orthopedic OPD with ankle joint-foot complex pathology. During the pilot period, around 263 patients with ankle joint-foot pathology attended the orthopedic OPD. A total of 100 patients, out of 250, were assessed for anthropometric measurements after receiving informed consent. Moreover, 11 patients were removed from the study due to a change in diagnosis, and two patients were denied informed consent. It was not feasible to evaluate every patient with ankle-foot pathology reporting to the OPD during the first phase for the arthrokinematic measurements. All 100 patients were selected by the random sampling method.

The sample size was revised based on a reference study (outside India) and the incidence of ankle joint-foot complex pathologies observed at the OPD of AIIMS Rajkot. In the continuation period (August 1, 2023, to February 28, 2024), 58 patients with ankle joint-foot pathology were assessed. The anthropometric data of the 158 patients was compiled in an Excel sheet, tabulated, graphically represented, and statistically analyzed. All 158 patients were selected by the random sampling method.

Table 1 shows that out of the total 158 patients with ankle joint-foot complex pathologies examined, 141 (89.24%) had low OA, i.e., occupations involving low physical activity. A statistically significant association of these musculoskeletal pathologies was observed with OA (P < 0.05).

**Table 1:** Distribution of musculoskeletal conditions of the ankle joint-foot complex based on the physical activity involved in the occupation Values are mentioned in the column as numbers, and % denotes the percentage proportion of those values out of the total sample. If the P-value is less than 0.05, then it is significant. OA, occupational activity

Ankle joint-foot pathologies	High OA	Low OA	Total (no.)
Sprain of the ankle and foot	08 (37.2%)	57 (32.6%)	65
Isolated ankle joint sprain	04 (10.2%)	34 (26.5%)	38
Isolated foot sprain	02 (0.0%)	14 (2.2%)	16
Plantar fasciitis	0 (3.8%)	09 (1.3%)	9
Ankle joint pain	02 (10.2%)	04 (5.7%)	6
Foot pain	0 (9.0%)	10 (7.4%)	10
Flat foot	1 (6.4%)	06 (2.2%)	7
Ankle and foot pain	0 (0.0%)	03 (4.3%)	3
Others	0 (0.0%)	04 (1.7%)	4
Total number of patients	17	141	158
Fisher’s exact test	P = 0.0482, S		

Table 2 shows a statistically significant difference in mean dorsiflexion, subtalar joint - inversion, and subtalar joint - eversion (P < 0.05). The mean measurements of dorsiflexion, subtalar joint - inversion, and subtalar joint - eversion were observed to be significantly lower in the affected foot as compared to the non-affected foot.

**Table 2:** Arthrokinematic measurements of variables with affected and non-affected feet FB, foot breadth; FL, foot length; LAA, longitudinal arch angle; MLAH, medial longitudinal arch height; NS, not significant; S, significant; TFL, truncated foot length If the P-value is >0.05, then it is statistically insignificant.

Variables	Side of foot	Mean ± SD	t-test	P-value
FL (mm)	Affected	233.29 ± 17.38	t = 0.055	P = 0.956, NS
	Non-affected	233.40 ± 17.35		
FB (mm)	Affected	88.13 ± 14.94	t = 0.034	P = 0.973, NS
	Non-affected	88.19 ± 14.81		
TFL (mm)	Affected	175.16 ± 14.96	t = 0.047	P = 0.963, NS
	Non-affected	175.08 ± 14.83		
MLAH (cm)	Affected	6.26 ± 1.54	t = 0.034	P = 0.973, NS
	Non-affected	6.27 ± 1.53		
LAA (degree)	Affected	154.86 ± 14.63	t = 0.786	P = 0.432, NS
	Non-affected	155.94 ± 9.40		
Dorsiflexion (degree)	Affected	19.11 ± 6.31	t = 1.986	P = 0.046, S
	Non-affected	20.49 ± 5.87		
Plantar flexion (degree)	Affected	39.32 ± 12.73	t = 1.783	P = 0.078, NS
	Non-affected	41.89 ± 12.24		
Subtalar joint - inversion (degree)	Affected	16.14 ± 5.48	t = 2.160	P = 0.032, S
	Non-affected	18.84 ± 5.27		
Subtalar joint - eversion (degree)	Affected	7.77 ± 2.95	t = 2.290	P = 0.23, S

**Figure 1:**
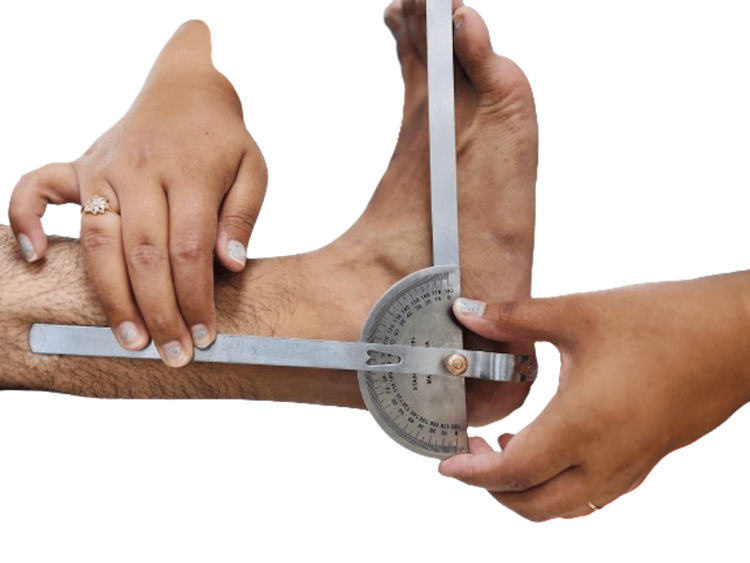
Dorsiflexion at the ankle joint Courtesy: The image was captured and edited by Dr. Valency Pateliya and Dr. Rajdip Herma, junior residents of the Department of Anatomy, AIIMS Rajkot.

Table 3 shows that the mean dorsiflexion in patients with high OA was seen as lower as compared to patients with low OA in the affected foot and non-affected foot, but statistically, the finding was significant (P < 0.05). Figure 1 displays the technique for measuring dorsiflexion.

**Table 3:** Analysis of dorsiflexion in affected and non-affected feet based on physical activity For the t-test, if the P-value is <0.05, it is significant. For the ANOVA test, if the P-value is <0.01, then it is significant. NS, not significant; OA, occupational activity; S, significant

Physical activity	Affected foot	Non-affected foot	Difference in mean (MD)	t-test value	P-value
	Mean ± SD	Mean ± SD			
High OA (n = 17)	18.64 ± 6.06	20.17 ± 4.79	1.53 ± 1.27	t = 2.471	P = 0.0237, S
Low OA (n = 141)	19.29 ± 6.26	20.41 ± 6.01	1.12 ± 0.25	t = 2.03	P = 0.041, S
Total	19.11 ± 6.17	20.34 ± 5.72	1.23 ± 0.45	t = 2.316	P = 0.0210, S
t-test and P-value	t = 0.406; P = 0.685, NS	t = 0.154; P = 0.878, NS	-	-	-

**Figure 2:**
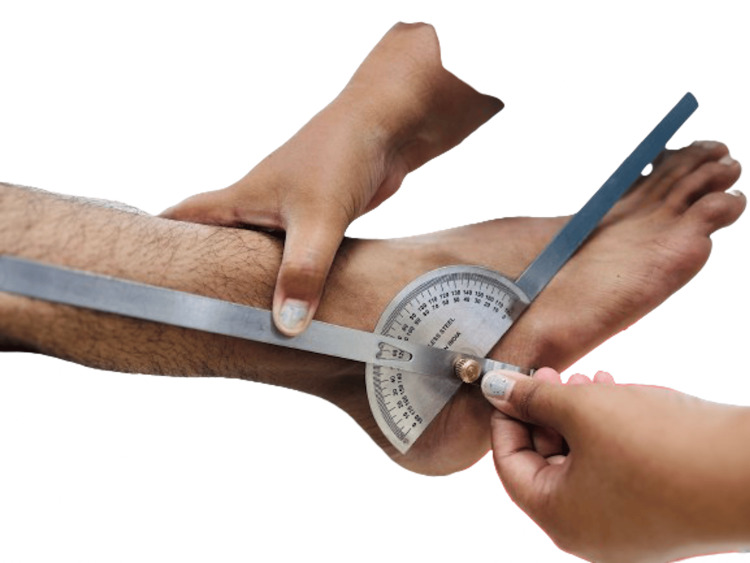
Plantar flexion at the ankle joint Courtesy: The image was captured and edited by Dr. Valency Pateliya and Dr. Rajdip Herma, junior residents of the Department of Anatomy, AIIMS Rajkot.

Table 4 reveals that the mean plantar flexion in patients with high OA was seen as lower as compared to that in patients with low OA in the affected foot. This finding was found to be statistically significant (P < 0.05). In the non-affected foot, the mean plantar flexion in patients with high OA was lower than in patients with low OA. This finding was statistically not significant (P > 0.05). Figure 2 displays the technique for measuring plantar flexion.

**Table 4:** Analysis of plantar flexion in affected and non-affected feet based on physical activity For the t-test, if the P-value is <0.05, it is significant. For the ANOVA test, if the P-value is <0.01, then it is significant. NS, not significant; OA, occupational activity; S, significant

Physical activity	Affected foot	Non-affected foot	Difference in mean (MD)	t-test value	P-value
	Mean ± SD	Mean ± SD			
High OA	37.94 ± 7.08	40.29 ± 5.14	2.35 ± 1.94	t = 2.721	P = 0.015, S
Low OA	39.74 ± 13.32	41.91 ± 12.77	2.17 ± 0.55	t = 3.163	P = 0.026, S
Total	39.12 ± 11.21	41.26 ± 9.07	2.14 ± 2.14	t = 2.931	P = 0.005, S
t-test and P-value	t = 0.548; P = 0.585, NS	t = 0.517; P = 0.606, NS	-	-	-

**Figure 3:**
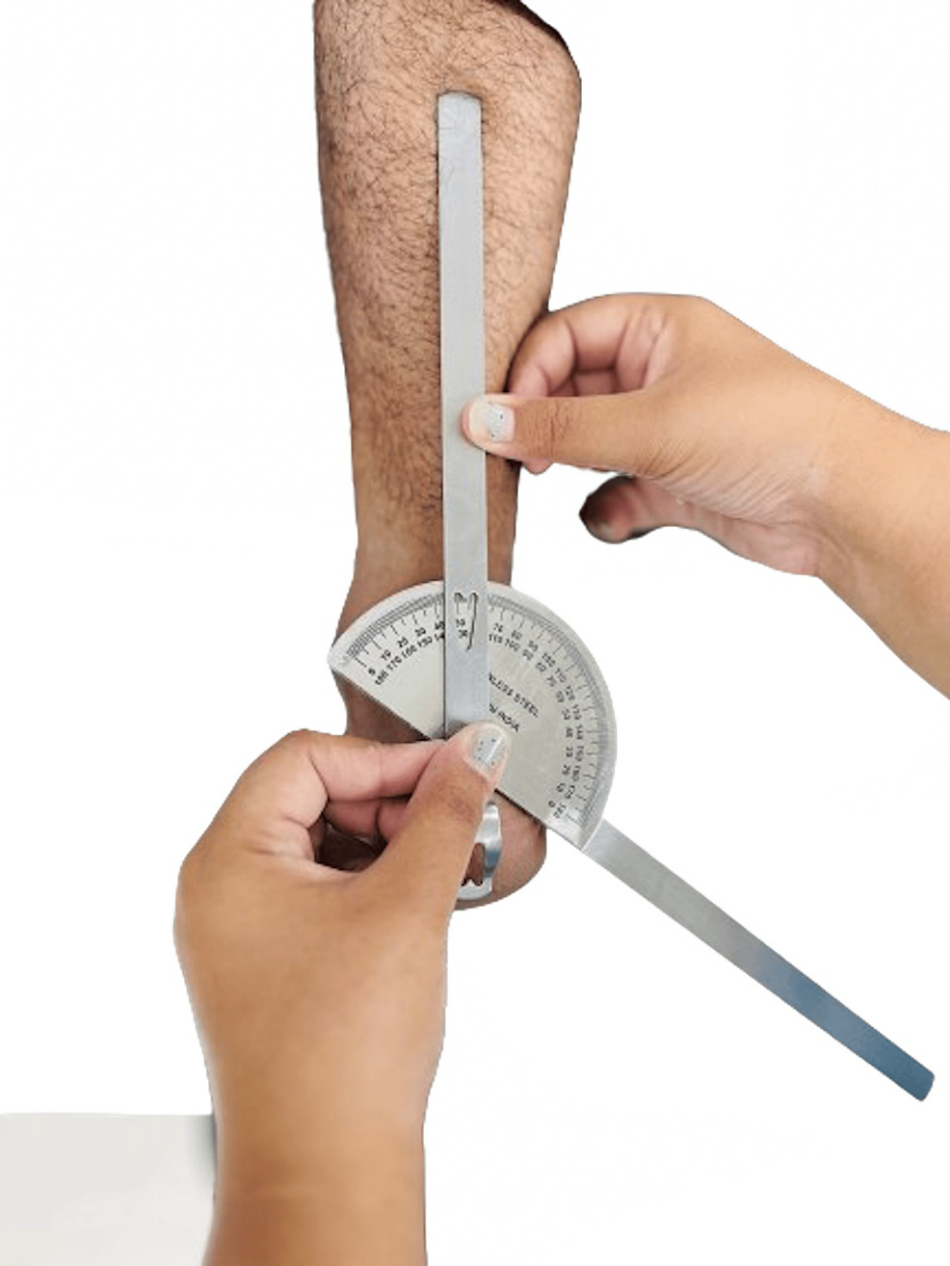
Inversion at the subtalar joint Courtesy: The image was captured and edited by Dr. Valency Pateliya and Dr. Rajdip Herma, junior residents of the Department of Anatomy, AIIMS Rajkot.

Table 5 shows that the mean inversion in patients with low OA was seen as lower compared to patients with high OA in the affected foot and non-affected foot, but statistically, the finding was not significant (P > 0.05). At the same time, there was a statistically significant difference in mean inversion between the affected foot and the non-affected foot in the high OA and low OA patients and both groups (P < 0.05). Figure 3 displays the technique for measuring inversion.

**Table 5:** Analysis of inversion in affected and non-affected feet based on physical activity For the t-test, if the P-value is <0.05, it is significant. For the ANOVA test, if the P-value is <0.01, then it is significant. NS, not significant; OA, occupational activity; S, significant

Physical activity	Affected foot	Non-affected foot	Difference in mean (MD)	t-test value	P-value
	Mean ± SD	Mean ± SD			
High OA	16.53 ± 3.46	17.53 ± 3.06	1.0 ± 0.40	t = 1.993	P = 0.046, S
Low OA	15.01 ± 3.98	16.02 ± 4.07	1.01 ± 0.09	t = 2.163	P = 0.038, S
Total	15.53 ± 3.73	16.68 ± 3.82	1.15 ± 0.05	t = 2.102	P = 0.040, S
t-test and P-value	t = 0.091; P = 0.621, NS	t = 0.177; P = 0.749, NS	-	-	-

**Figure 4:**
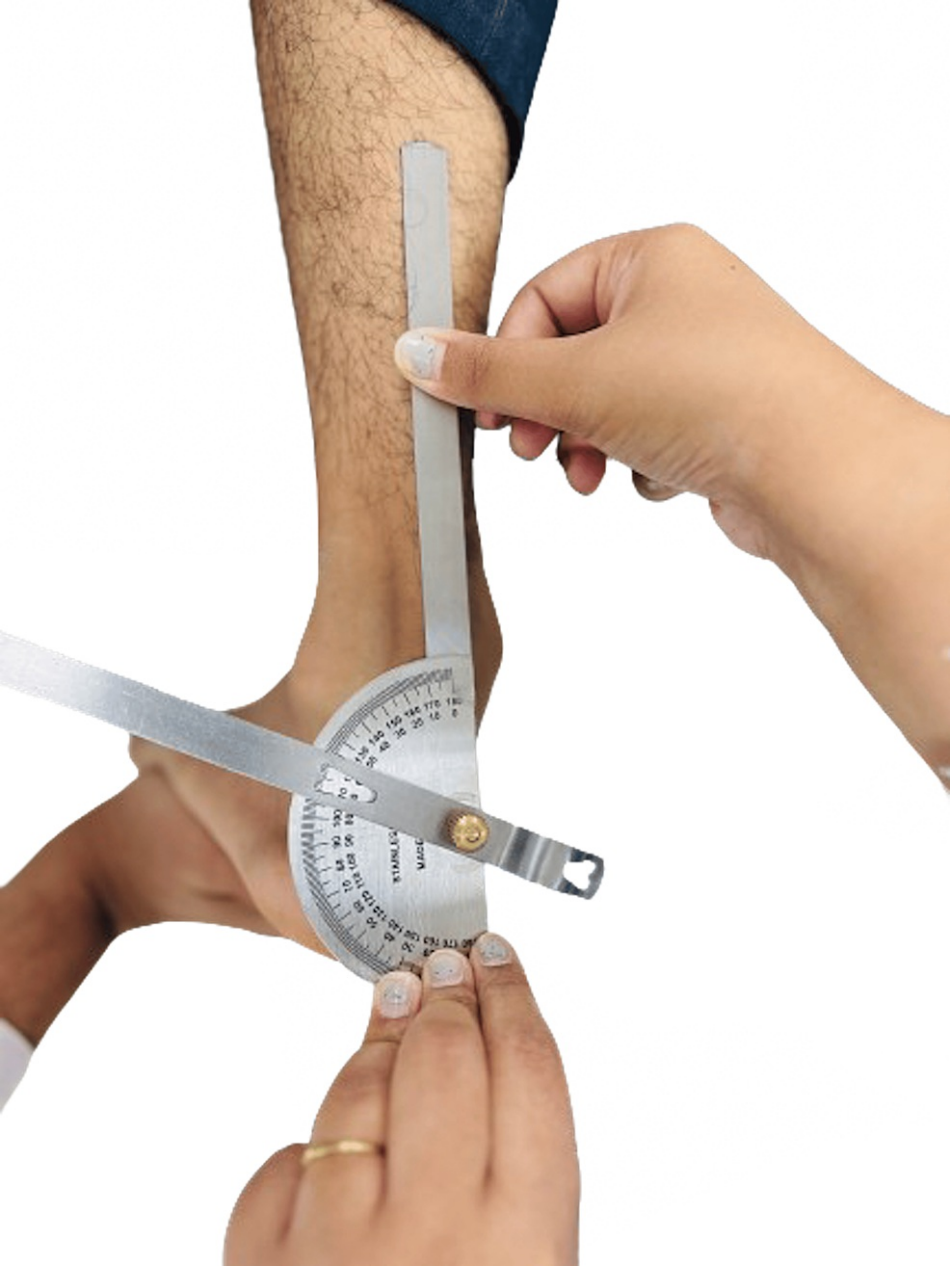
Eversion at the subtalar joint Courtesy: The image was captured and edited by Dr. Valency Pateliya and Dr. Rajdip Herma, junior residents of the Department of Anatomy, AIIMS Rajkot.

Table 6 reveals that the mean eversion in patients with high OA was seen as lower as compared to that in patients with low OA in the affected foot. Non-affected foot mean eversion in patients with high OA was higher than in patients with low OA. The findings were statistically not significant (P > 0.05). Figure 4 displays the technique for measuring eversion.

**Table 6:** Analysis of eversion in affected and non-affected feet based on physical activity For the t-test, if the P-value is <0.05, it is significant. For the ANOVA test, if the P-value is <0.01, then it is significant. NS, not significant; OA, occupational activity; S, significant

Physical activity	Affected foot	Non-affected foot	Difference in mean (MD)	t-test value	P-value
	Mean ± SD	Mean ± SD			
High OA	7.20 ± 2.17	8.00 ± 2.23	0.8 ± 0.06	t = 1.521	P = 0.107, NS
Low OA	7.28 ± 2.83	7.69 ± 2.95	0.41 ± 0.12	t = 0.741	P = 0.681, NS
Total	7.26 ± 2.63	7.93 ± 2.71	0.67 ± 0.08	t = 1.183	P = 0.271, NS
t-test and P-value	t = 0.109; P = 0.913, NS	t = 0.383; P = 0.703, NS	-	-	-

## Discussion

Ahlberg et al. stated that joint mobility varies from race to race [19]. For example, Negroes and Indians have a greater ROM than Caucasians [20]. Several researchers, namely Szabó et al., James and Parker, Nigg et al., and Nitz and Low-Choy, in their respective studies, described that the ROM decreases with age, particularly dorsiflexion-plantar flexion occurring at the ankle joint and inversion-eversion at the subtalar joint, which is 12-30% lower in older people [21-24]. We observed similar findings in our study.

In the present study, the ROM at the ankle joint and foot, especially the mean measurements of dorsiflexion, subtalar joint - inversion, and subtalar joint - eversion was significantly lower in the affected foot as compared to the non-affected foot.

The mean ROM, especially inversion and eversion at the subtalar joint, was observed to be higher in the non-affected feet of the patients with high OA compared to those with low OA. This observation indicates that in patients with high OA, like farmers and laborers, the osseoligamentous structures (bones, muscle tissue, tendons and ligaments, and skin) of the ankle joint-foot complex are more dynamically flexible compared to patients with low OA. This has proven our initial hypothesis correct. In their cross-sectional investigation encompassing 144 participants, Boukabache et al. established for the first time an association between passive hip extension and prolonged sitting or physical inactivity, potentially indicating a physiological adaptation in passive muscle stiffness. They further proposed that prolonged inactivity could be a cause of musculoskeletal pain [25].

The mean difference in the ROM, i.e., dorsiflexion, plantar flexion, inversion, and eversion, between affected and non-affected feet was found to be lower in the patients who belonged to low OA compared to those with high OA. This again indicates that in patients with low OA or those with sedentary lifestyles, the flexibility of the osseoligamentous structures of the ankle joint-foot complex is reduced. The reason could be that a less active lifestyle results in the weakening of ankle and foot muscles and the tightening of the tendons of the posterior compartment of the leg, like the soleus and gastrocnemius, required for flexion of the ankle joint. In their 2022 study involving 80 healthy adults, Dragoi et al. similarly stated that the static posture associated with sitting led to a more pronounced decrease in average peak ankle torque compared to active behavior [26].

Limitations of the study

The arthrokinematic measurements were taken using digital vernier calipers and goniometers by two of the investigators, SC and AKM. Intra-subject variability was identified by using only the radiographs that were already taken by the patients. Phase 2 of this study involves a comprehensive radiographic assessment of patients recorded in Phase 1.

## Conclusions

Our study underscores the significant impact of reduced physical activity on the ankle joint-foot complex, particularly in cases of sprains and related injuries within the Indian population. The findings highlight how decreased physical activity can lead to stiffness and diminished flexibility in the tendons, ligaments, and muscles surrounding the ankle joint-foot complex, notably affecting inversion and eversion movements. Moreover, our research indicates that individuals exposed to prolonged lower levels of physical activity may experience suboptimal recovery in terms of biomechanical function compared to their pre-injury levels. These conclusions emphasize the critical importance of maintaining appropriate physical activity levels to facilitate optimal recovery and function following ankle and foot injuries. Such insights can inform tailored rehabilitation strategies aimed at restoring flexibility and ROM, thereby promoting more effective recovery outcomes in affected individuals.
